# A New Wax-Holder for Taking Impressions of the Mouth

**Published:** 1848-01

**Authors:** 


*A New Wax-holder for taking Impressions of the Mouth.*—We are indebted to G. F. J. Colburn, Dentist of Morristown, N. J., for an improved wax-holder, for taking impressions of the mouth, which he has had the kindness to send to us, and for which, we tender him our thanks. We have not yet had an opportunity of using it, and cannot, therefore, speak from experience of its advantages. The difference between it and those in common use is, that it has two rims attached to the outer margin of the plate and instead of describing a half circle, its extremities are turned out. The outer rim is intended to protect the wax against injury from the cheeks and corners of the mouth in removing the impression. The author of this improvement says, he has often succeeded in procuring an accurate impression with this frame, after having failed with those in common use. After we shall have tried it, we may, perhaps, be able to say more concerning it.—*Balt. Ed.*

